# Incidence rate and sex ratio in multiple sclerosis in Lithuania

**DOI:** 10.1002/brb3.1150

**Published:** 2018-11-28

**Authors:** Daiva Valadkeviciene, Andrius Kavaliunas, Rasa Kizlaitiene, Mykolas Jocys, Dalius Jatuzis

**Affiliations:** ^1^ Department of Neurology and Neurosurgery, Faculty of Medicine, Institute of Clinical Medicine Vilnius University Vilnius Lithuania; ^2^ Department of Clinical Neuroscience Karolinska Institute Stockholm Sweden; ^3^ Faculty of Medicine Vilnius University Vilnius Lithuania

**Keywords:** age‐groups, demography, epidemiology, forecasting, incidence, multiple sclerosis, population dynamics, sex ratio

## Abstract

**Objectives:**

To determine the temporal changes in incidence rates of multiple sclerosis (MS) over the past 15 years in Lithuania with prediction up to 2020, and to estimate female‐to‐male sex ratio and its changes among MS patients.

**Materials and Methods:**

We conducted a descriptive incidence study. The crude incidence rates (CIR) were calculated using 15‐year period, sex, age‐groups, and the number of newly registered MS patients. Standardized incidence rates (SIR) were calculated using European standard in order to evaluate the influence of resident structure changes on incidence of MS during the last 15 years. The data were processed using Minitab set to estimate a linear trend model for the temporal changes of 16 parameters.

**Results:**

The data showed a substantial growth of the incidence rate of MS in Lithuania during the period of 2001–2015. In 2001, MS was diagnosed to 162 new individuals, whereas 343 new cases of MS were diagnosed in 2015. During 2001–2015, the incidence of MS was on average 6.5 (95% CI 5.70–7.30) cases per 100,000 residents, and 4.9 (95% CI 4.46–5.34) and 8.1 (5.86–9.34) for 100,000 male and female, respectively. Female‐to‐male sex ratio in MS in Lithuania had a tendency to increase over the period. Females were affected from 1.5 to 2 times more often than males.

**Conclusions:**

In 2020, the incidence rate of MS is estimated to reach 13 cases per 100,000 persons and females are expected to be diagnosed with MS two times more often than males.

## 1 INTRODUCTION

Multiple sclerosis (MS) is a chronic autoimmune demyelinating disease of the central nervous system. During its clinical course, various neurodegenerative and autoimmune processes are known to be taking place; however, its etiology is unknown.

For many years, MS has been considered to be more prevalent among females and in the regions further from the Equator (Wiese, Rodriguez Escobar, Hsu, Kulathinal, & Hayes‐Conroy, 2017). However, the recent studies show that the latitude gradient is diminishing and that the female‐to‐male sex ratio in MS patients has increased over the past decades (Wiese et al., 2017).

Approximately 2.5 million people worldwide are affected with multiple sclerosis (MS) that is a common cause of serious physical disability in young adults (Compston & Coles, 2008; Swingler & Compston, 1988). In the northern parts of North America and Europe, where the disease is most common, the prevalence is approximately 0.1%–0.2% of the population (i.e., 100–200 per 100,000 population) and the incidence is approximately 5–6 per 100,000 population per year (Goodin, 2014).

An absolute majority of researchers indicate that the incidence of MS is on a sharp rise (Grimaldi et al., 2007). This has been supported by studies carried out in Norway (Larsen, Kvaale, Riise, Nyland, & Aarli, 1984), Italy (Granieri et al., 2000), United Kingdom (Alonso, Jick, & Olek, 2007), Sweden (Svenningsson, Runmarker, Lycke, & Andersen, 1990), Finland (Kinnunen, 1984), and many other countries. The problem with the most of the previously mentioned studies is that the incidence estimates do not cover the whole country, which is why it is so difficult to confirm the increase in incidence on a national level. Some meta‐analyses claim that the overall increase in incidence may be related to subjective factors such as improved diagnostics and registry (Browne et al., 2014; Kingwell et al., 2013). Improved conditions of incidence research on a population‐based level would offer new possibilities for more objective studying of MS incidence dynamics.

The research on temporal changes of incidence rates in MS in Lithuania is scarce; however, according to studies, Lithuania, together with other Baltic states, remains a region with higher MS incidence rate compared to other nations (Pugliatti et al., 2006), with more than 2,000 confirmed MS cases in a country with 2.9 million inhabitants.

Our goal in this study was to determine the temporal changes in incidence rates of MS over the past 15 years (between 2001 and 2015) in Lithuania, to estimate female‐to‐male sex ratio in MS and its changes over the years, and to predict incidence up to 2020.

## 2 MATERIALS AND METHODS

A descriptive incidence study was conducted, obtaining and analyzing the data from the Compulsory Health Insurance Information System “Sveidra” (CHIIS) held by the National Health Insurance Fund. This system registers every disease diagnosed to the patient during ambulatory checkups and stationary visits. CHIIS data provide a possibility to analyze information on an individual level, eliminate the duplicate diagnoses, and calculate the health indicators according to the patient's residency.

The resident number in sex and age‐group categories was obtained from the Statistics Lithuania (a national agency that develops, produces, and disseminates official statistics). The study investigated the population of Lithuania. The variables used for analysis were as follows: years (from 2001 till 2015 [included]), sex (male and female), age‐groups, and the number of newly registered MS patients according to previously mentioned variables.

The crude incidence rates (CIR—ratio between individuals with newly diagnosed MS in ambulatory service according to International Classification of Diseases version 10 [ICD‐10] code G35 and mean yearly resident number) were calculated. In order to evaluate the influence of resident structure changes on incidence of MS during the last 15 years, the direct method of standardization using European standard was applied and standardized incidence rates (SIR) were calculated. 95% confidence intervals were estimated for the averages. The data were processed using Minitab set to estimate a linear trend model for the temporal changes of 16 parameters. The quality of equating the dynamic curve was evaluated using three additional and each other complimenting precision measures: mean absolute percentage error (MAPE), which counts the dynamic line equation in percentage; mean absolute deviation (MAD), which measures the precision of equated points (measured in the same units as the parameters we analyze); and MSD (mean squared deviation). The three mentioned measures allowed comparison of real values with theoretical values which were obtained using linear trend model. For the prediction of incidence, a simple trend extrapolation method was used from the linear regression line.

The annual average percent change (AAPC) was calculated using the equation:

$$\text{AAPC}=\left(\sqrt[{n-1}]{Y_{L}/Y_{F}-1}\right)\times 100\%$$

where *n* is the number of years, *Y*_L_ is the last, and *Y*_F_ is the first calendar year theoretical incidence rate.

## 3 RESULTS

The data from CHIIS showed that the number of newly diagnosed MS cases was increasing in Lithuania every year. In 2001, MS was diagnosed to 162 new individuals (61 male and 101 female), whereas 343 new cases of MS (101 male and 242 female) were diagnosed in 2015. The CIR and SIR of MS are presented in Table 1. During 2001–2015, the incidence of MS was on average 6.5 (95% CI 5.70–7.30) cases per 100,000 residents, and 4.9 (95% CI 4.46–5.34) and 8.1 (95% CI 6.86–9.34) for 100,000 male and female, respectively. These rates had been fluctuating over the past 15 years—the difference between the highest and the lowest incidence rate was 5.7 times. The incidence difference among males was 2.9 times and among females 8.9 times.

**Table 1:** The crude and standardized incidence rates and sex ratios of multiple sclerosis in Lithuania during 2001–2015

	CIR	SIR	CIR for males	SIR for males	CIR for females	SIR for females	Crude sex ratio	Standardized sex ratio
Average	6.5	6.4	4.9	4.7	8.0	8.1	1.636	1.721
95% CI	5.70–7.30	5.62–7.18	4.46–5.34	4.28–5.12	6.82–9.18	6.86–9.34	1.475–1.797	1.539–1.903
Rank	5.71	5.47	2.92	2.79	8.75	8.95	0.98	1.10
Minimum	4.62	4.57	3.58	3.42	4.85	4.92	1.11	1.13
Maximum	10.33	10.04	6.50	6.21	13.60	13.87	2.09	2.23

CI: confidence intervals; CIR: crude incidence rate; SIR: standardized incidence rate.

**Figure 1:**
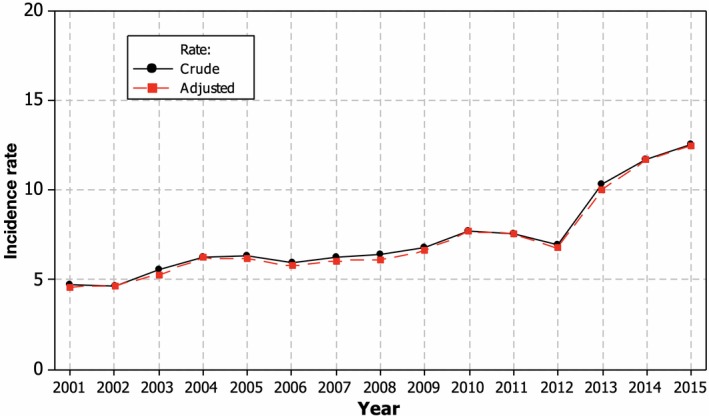
The dynamics of the incidence of multiple sclerosis in Lithuania 2001–2015

The comparison of CIR and SIR in the overall dynamics showed that changes in the age structure of the population during 2001–2015 had no significant influence (Figure 1). That is why we used CIR in the following analysis of dynamics of MS in Lithuania.

**Figure 2:**
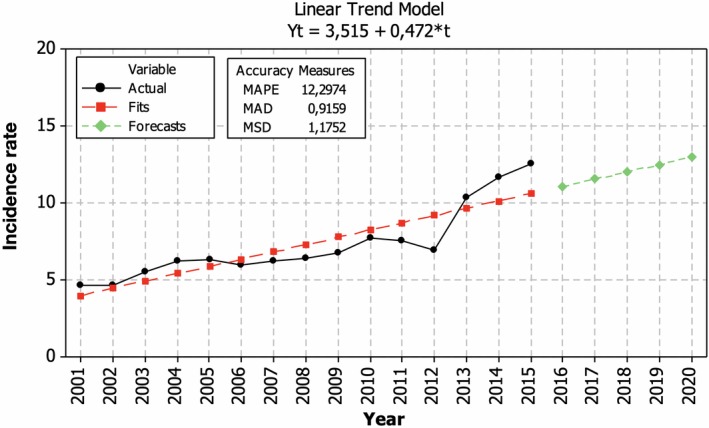
Temporal changes in the incidence of multiple sclerosis in Lithuania during 2001–2015 and its prognosis for 2020 (cases per 100,000 persons)

In Lithuania, the overall incidence rate during 2001–2015 tended to increase (Figure 2). A clear increase in the incidence rate of MS was registered in 2012, when the registration system of the diseases was changed. During the period of 2001–2015, the overall incidence rate was increasing on average 3.5 cases (7.4%) per 100,000 persons yearly. If the tendencies prevail, the overall incidence rate of MS is estimated to reach thirteen cases per 100,000 persons in 2020.

**Figure 3:**
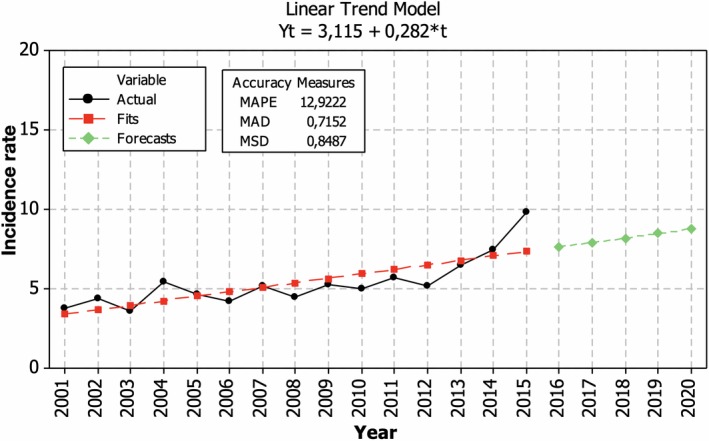
Temporal changes of sex‐specific incidence rate of multiple sclerosis for male in Lithuania during 2001–2015 and prognosis for 2020 (cases per 100,000 persons)

The incidence of MS among male showed only slight increase until 2012 and a sharp increase beginning 2013 (Figure 3). The incidence rate increased by 3.1 cases (5.6%) per 100,000 men yearly. If the tendencies remain, the incidence is estimated to reach nine cases per 100,000 males in 2020.

**Figure 4:**
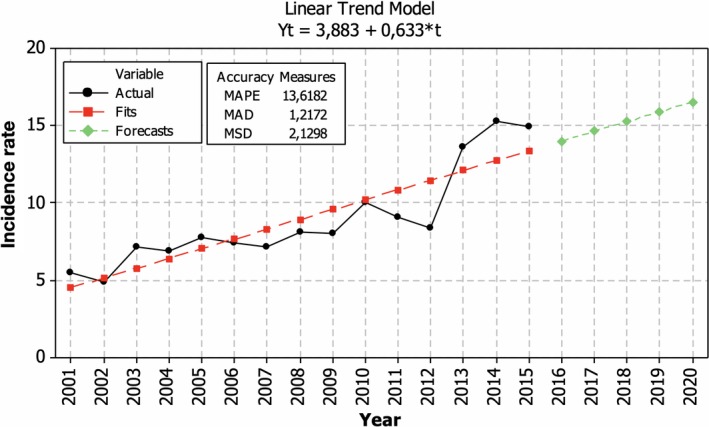
Temporal changes of sex‐specific incidence rate of multiple sclerosis for females in Lithuania during 2001–2015 and prognosis for 2020 (cases per 100,000 persons)

Temporal changes in the incidence rate in MS among female in Lithuania 2001–2015 showed a consistent growth in incidence up till 2010, a slight drop in 2011–2012, and a sharp rise from 2012 till 2014 (Figure 4). During the period of 2001–2015, incidence increased by 3.8 cases (8.1%) per 100,000 females yearly. If the tendencies remain, MS incidence among females in Lithuania is estimated to reach 16 new cases per 100,000 females in 2020.

**Figure 5:**
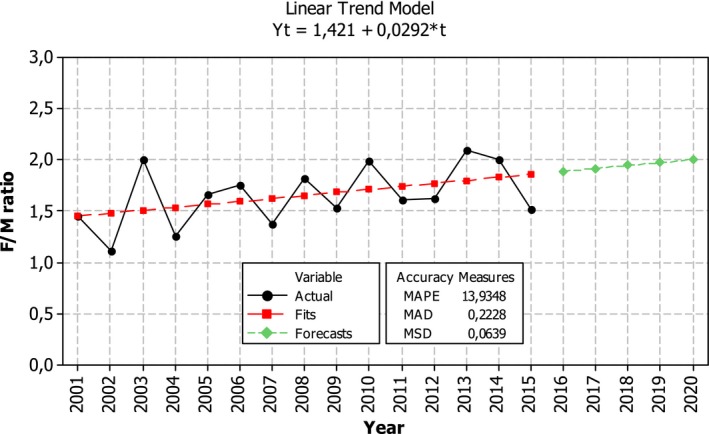
Temporal changes in Lithuanian female‐to‐male ratio in incidence rate in multiple sclerosis during 2001–2015 and its prognosis for 2020

Female‐to‐male sex ratio in the incidence rate in MS in Lithuania showed a tendency to increase over the period of 2001–2015. Currently, females are affected from 1.5 to 2 times more often than males. If this tendency remains in 2020, females are expected to be diagnosed with MS two times more often than males (Figure 5).

## 4 DISCUSSION

In this study of temporal changes of incidence rate and sex ratio of MS in Lithuania, we found that the incidence rate of MS in Lithuania increased by approximately 7.4% per year between 2001 and 2015 in both males and females. An overall incidence rate in MS has been slightly increasing up till 2010 and then dropped during the following 2 years. The increase in incidence rate in MS in 2012 was associated with changes within disease registry system. Starting June 1, 2011, comorbidities were included in the registry during stationary visits. Also, in 2013 a new and adjusted coding was introduced for ambulatory patients at emergency departments. That is why there has been a sharp rise in number of new patients, as well as comorbidities, during the previous years that reflect an earlier underreporting, which has been later adjusted for. Analysis of temporal changes of simple and age‐standardized general incidence rates showed that the changes in the age structure of 5‐year age‐groups had no significant influence to the incidence rate of MS comparing to other chronic diseases (such as cardiovascular and epithelial tumors).

Our prediction of MS incidence for 2020—13 cases per 100,000 persons—should be regarded with caution due to several important reasons. First, as many other prediction estimates, ours is also subjected to a ceteris paribus assumption, essentially meaning “all other things being equal,” or as we do note in the article—if tendencies prevail. As mentioned earlier, it is highly dependent on the registration system itself and the amendments the system undergoes from time to time. Besides the changes within disease registry system, it is important to note that changes of the diagnostic criteria may have an influence on the commonly used measures of disease frequency in epidemiology. Due to McDonald^'^s criteria revisions in 2005 and 2011, early diagnosis of MS became possible due to updates of magnetic resonance imaging assessment. In addition, diagnosis was improved due to improved access to information about clinical and paraclinical MS symptoms to public, neurologists, and other medical practitioners. Further changes can be associated with a more active monitoring, starting with a conversion from a clinically isolated syndrome to MS. Changes in lifestyle behavior and other known environmental risk factors, such as vitamin D and smoking, might have also impacted the incidence of MS, but this needs to be explored furthermore, as we aimed for a descriptive study, rather than establishing associations.

Our study is in line with the recent studies reporting the increasing incidence of MS. Results of meta‐analyses suggest that the incidence of MS has increased over time and provide some evidence that this has primarily resulted from an increase in the incidence of MS among women (Alonso & Hernan, 2008; Koch‐Henriksen & Sørensen, 2010; Orton et al., 2006; Ramagopalan et al., 2010; Trojano et al., 2012). The recency of this increase suggests that changing environmental or lifestyle factors is interacting with biological sex to increase MS risk predominantly in females. Indeed, a number of recent studies have identified sex‐specific differences in the effect of environmental factors on MS incidence (Dunn, Gunde, & Lee, 2015). According to a latest systematic review (Kingwell et al., 2013), incidence estimates tended to be higher in the Northern regions of the United Kingdom and in the Nordic Countries, implicating the role of latitude. This pattern is not uniform, however, with higher estimates originating as far south as Sicily and Greece. The incidence sex ratios revealed consistently higher rates of women than men with MS across Europe with no obvious patterns between north and south. For unknown reasons, approximately three quarters of people with multiple sclerosis are female, as is common in diseases that are considered autoimmune (Reich, Lucchinetti, & Calabresi, 2018).

While much of the literature has focused on specific regions or individual cities within a given country, a few studies reported countrywide data, which is without no doubt one of the strengths of the current study. To add, this study gives an insight of MS epidemiology into the Baltic region, as there is scarce of the reliable data and epidemiologic measures reported either from Lithuania, or Latvia, or Estonia.

The distribution and frequency of MS, assessed by estimate of incidence, provide essential information for health service planning and can be used to monitor or reveal spatial, temporal, and demographic differences in the distribution of disease.

## 5 CONCLUSIONS

This study highlights substantial growth of the incidence rate of MS in Lithuania during the period of 2001–2015. The findings show that while both male and female incidence rates have a strong tendency to rise, females are expected to be diagnosed with MS two times more often than males in 2020. Further research should report and analyze comparative indices between neighboring nation states and evaluate socioeconomic differences in MS incidence and prevalence.

## CONFLICT OF INTEREST

None declared.
